# Machine Learning Aided Scheme for Load Balancing in Dense IoT Networks

**DOI:** 10.3390/s18113779

**Published:** 2018-11-05

**Authors:** Cesar A. Gomez, Abdallah Shami, Xianbin Wang

**Affiliations:** Department of Electrical and Computer Engineering, Western University, London, ON N6A 5B9, Canada; cgomezsu@uwo.ca (C.A.G.); abdallah.shami@uwo.ca (A.S.)

**Keywords:** Internet of things (IoT), smart cities, heterogeneous networks (HetNets), load balancing, machine learning, Markov Decision Process (MDP), LoRaWAN

## Abstract

With the dramatic increase of connected devices, the Internet of things (IoT) paradigm has become an important solution in supporting dense scenarios such as smart cities. The concept of heterogeneous networks (HetNets) has emerged as a viable solution to improving the capacity of cellular networks in such scenarios. However, achieving optimal load balancing is not trivial due to the complexity and dynamics in HetNets. For this reason, we propose a load balancing scheme based on machine learning techniques that uses both unsupervised and supervised methods, as well as a Markov Decision Process (MDP). As a use case, we apply our scheme to enhance the capabilities of an urban IoT network operating under the LoRaWAN standard. The simulation results show that the packet delivery ratio (PDR) is increased when our scheme is utilized in an unbalanced network and, consequently, the energy cost of data delivery is reduced. Furthermore, we demonstrate that better outcomes are attained when some techniques are combined, achieving a PDR improvement of up to about 50% and reducing the energy cost by nearly 20% in a multicell scenario with 5000 devices requesting downlink traffic.

## 1. Introduction

Thanks to the proliferation of Internet-connected wireless devices, the Internet of things (IoT) and the machine-to-machine (M2M) communications paradigms, highly dense cellular networks have emerged as a connectivity solution for large scale IoT applications. These wireless devices are diverse and comprise not only increasingly powerful devices like smart phones, but also tiny ones such as sensors, actuators, wearable electronics, etc. To alleviate the congestion in dense wireless networks, a number of solutions have been proposed. For instance, the idea of heterogeneous networks (HetNets) has been conceived. In a HetNet, the network infrastructure is supported by heterogeneous elements consisting of macro base stations (MBS), which provide a wide area coverage, and small base stations (SBS), that are meant to cover high traffic hotspots. The design of a cellular HetNet is based on a multi-tier topology, which features overlapped coverage between a tier of MBS and several subtiers of SBS. This design enhances the network capacity but at the cost of a challenging co-existence governing the network topology [1]. In fact, in urban areas, more SBS are added each year to the existing networks, creating a HetNet scenario where a wireless device may communicate with multiple BS, either MBS or SBS [2].

One of the most challenging design issues in HetNets is to achieve an optimal load balance among the base stations (BS), since the network traffic might be unevenly distributed. To this end, the association between devices and serving BS is a critical consideration. In homogeneous wireless networks, like the traditional cellular networks, a device is associated with the BS providing the strongest signal and, therefore, the association mechanisms are based on metrics such as signal-to-noise ratio (SNR) or received signal strength indicator (RSSI). However, this association method is not efficient for HetNets in terms of network capacity, since other critical aspects should be considered, such as, for example, the traffic load on the BS to be associated [3]. Device association methods based on signal metrics may lead to a major load imbalance in HetNets because MBS usually offer higher transmit power to devices than SBS. Consequently, load balancing methods for HetNets have been proposed by considering performance metrics like outage/coverage probability, spectrum efficiency, energy efficiency, uplink-downlink asymmetry, backhaul bottleneck, and mobility support [4]. Nevertheless, the achievement of a balanced HetNet is not easy and intelligent mechanisms that consider the traffic load and all related network conditions of BS are desirable due to the overall complexity of the process [5]. For this reason, artificial intelligence theory has been applied to overcome these kinds of challenges in complex systems like HetNets.

Load balancing in a HetNet may be performed by using either a single radio access technology (RAT) or multiple RAT (Multi-RAT). Multi-RAT techniques are aimed at taking advantage of load balancing between spectrum licensed technologies, e.g., cellular networks, and unlicensed ones, e.g., WiFi. However, the RAT selection algorithms, as well as the offloading mechanisms across cellular BS and WiFi access points, comprise an ambitious goal in terms of coordination and quality of service (QoS) [6]. In this work, we focus on the load balancing problem by considering a single RAT and its application to an actual IoT network. Specifically, the RAT used in this study is the LoRaWAN (long-range wide-area network) standard.

LoRaWAN is one of the most notable LPWAN (low-power wide area-network) technologies, alternative standards to conventional cellular networks, which have noteworthy expansions through IoT services providers [7]. As with other LPWAN technologies, LoRaWAN devices operate at a very low power, with long coverage (end devices can connect to a BS at a several-kilometers distance), and through a star topology, such as cellular networks [8,9]. Another important characteristic is that LoRaWAN works in the unlicensed sub-GHz band, which is suitable for IoT applications in complex environments. However, the LoRaWAN protocol poses relevant challenges for dense networks regarding scalability and capacity. For example, in the default and most used class operation (Class A), LoRaWAN devices employ an uncoordinated access scheme (ALOHA) which might produce a collision avalanche in a large-scale network [10]. Therefore, optimization techniques are needed to allow reliable services and to avoid capacity drain in LoRaWAN networks with high densities of devices, such as those deployed in urban scenarios for smart cities.

In this paper, we first show that an urban LoRaWAN network may be deemed as a HetNet. Hence, we address the problem of load balancing in a HetNet through appropriate machine learning (ML) techniques and we apply the proposed solution to improve the performance of a LoRaWAN network in a city. We further evaluate the performance of our solution in terms of the packet delivery ratio (PDR) and energy cost of data delivery (ECD) when the network has from a few to several thousands of end devices connected to it. Moreover, we expand our analysis to the case when devices request downlink traffic and not only the basic IoT scenario where uplink traffic is analyzed. The evaluation of our scheme is based on data collected from an actual network and its results illustrate that both PDR and energy cost are enhanced.

In the next sections of this paper, we review relevant works related to load balancing methods in HetNets (Section 2); we explain the factors to consider for an urban LoRaWAN network as a HetNet (Section 3); we describe our proposed scheme and its methods (Section 4); we give details about our network simulation design (Section 5); and we present the evaluation results (Section 6). Finally, we conclude our work and discuss some possible future directions (Section 7).

## 2. Related Work

A variety of approaches exists in the literature regarding the single RAT load balancing in HetNets. One of the most studied techniques is the cell range expansion (CRE): a mechanism to virtually expand an SBS range by adding a bias value to the power that a device receives from that SBS. In this way, instead of increasing the actual transmit power of an SBS, a virtual range expansion is performed so that a device will not connect to an MBS, but an SBS. However, to find an optimal bias value for minimizing the devices’ outage is a non-trivial problem and depends on several factors.

Accordingly, in [11] a scheme is proposed for the bias value optimization based on the Q-learning algorithm. The authors show that their method can decrease the number of outage devices and improve average throughput compared to non-learning schemes with a common bias value. Conversely, in [12] Ye et al. present a load-aware association method applied to CRE by considering two types of biasing factors, signal-to-interference-plus-noise ratio (SINR) and rate. The authors point out that the optimal biasing factors are nearly independent of the BS densities across tiers, but highly dependent on the per-tier transmit powers. Authors in [13] develop a clustering algorithm to classify BS into groups and present a central-aided distributed algorithm for adjusting the CRE bias. Their objective is to obtain a solution for the rate-related utility optimization problem based on local information. Thus, a central MBS is used to collect the information from the SBS, which determine their own CRE bias based on the shared central information. Similarly, authors in [14,15] propose clustering techniques for optimizing the load balancing problem in HetNets.

Taking into account the energy efficiency, Ref. [16,17] present techniques that are basically based on active/sleep schemes for multitier HetNets. In a similar manner, Muhammad et al. propose in [18] an association method that selectively mutes certain SBS. Then, end devices are covered by CRE for achieving load balancing in non-uniform HetNets, i.e., networks with SBS randomly deployed close to the edges of the MBS coverage, where the signals are weak. Contrary to the uniform case, their results show that biasing has distinct effects on the coverage and rate performance of a non-uniform HetNet. Lastly, authors in [19] propose a load balancing solution for a two-tier HetNet based on stochastic geometry. Their algorithm performs a CRE biasing to achieve an optimal SBS density regarding network energy efficiency.

Overall, biasing methods such as CRE are aimed at finding the appropriate bias values and at determining whether a specific BS should be considered or not for communication with a particular wireless device. An optimal decision of this association yields a network with balanced BS. This enhances the network performance in terms of capacity and energy efficiency, for instance, especially in scenarios with a large number of devices.

Although several ML algorithms have been presented in the literature to address the load balancing problem, they mainly focus on reinforcement learning techniques. Our method uses an unsupervised technique to discover the hidden pattern behind the selected features and a supervised technique to take advantage of the historical labeled data. Then, a supervised classifier is applied in order to accomplish a biasing scheme by contemplating metrics that are not directly related to signals strength. In this way, our model learns from data to predict a device-BS association without considering signal-based measurements. Additionally, our method employs a Markov Decision Process (MDP) to determine whether a BS needs to be balanced or not. For both techniques, the data are obtained from a real IoT LoRaWAN network deployed in an urban area, which is the use case scenario for our solution. To the best of our knowledge, this paper is the first one that presents a solution to the load balancing problem applied to a LoRaWAN network.

## 3. A LoRaWAN Network Seen as a HetNet

As we have explained, the BS in a HetNet are dissimilar in terms of coverage and, therefore, BS are either MBS or SBS. We have also mentioned that LoRaWAN networks are cellular-like and are deployed following a star topology. However, unlike traditional cellular networks, LoRaWAN is an open standard and operates in the unlicensed bands, which allows rapid implementation of public and private networks. Then, in a smart city scenario where the priority of an IoT network might be capacity rather than communication range, the LoRaWAN access points are prone to being deployed in a non-homogeneous manner.

**Figure 1:**
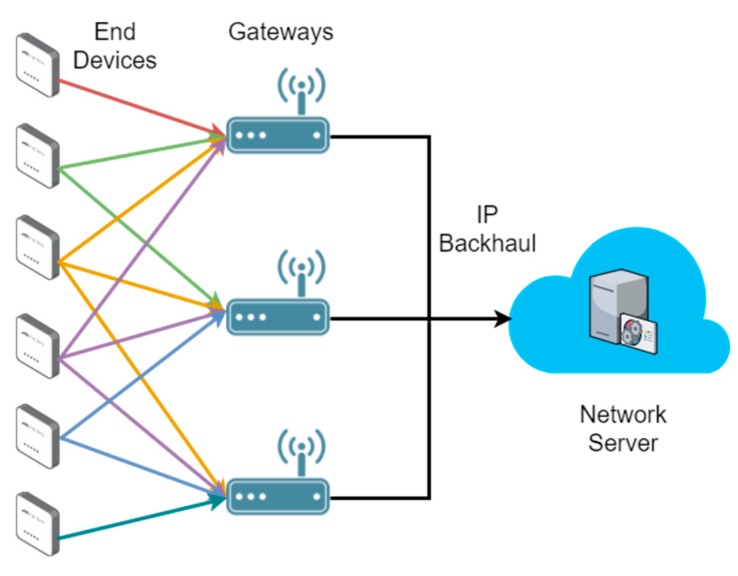
LoRaWAN network architecture. An end device may be associated with more than one gateway. Adapted from [21].

Moreover, it is also important to highlight that the LoRaWAN standard lets an end device be concurrently associated with more than one BS (i.e., gateway) [20], as shown in Figure 1. We take into account this characteristic to evaluate the performance of our load balancing scheme. In this way, to be consistent with the standard, our goal is to determine what BS should transmit the downlink (DL) message to an end device, once an uplink (UL) message is received through more than one BS. This procedure is not defined by the LoRaWAN specifications and a network operator has to choose an optimal mechanism for it. Therefore, we consider a number of Class A end devices transmitting confirmed UL packets, i.e., packets that need to be acknowledged (ACK), and a network server that must make decisions on which gateways should relay the DL packets to end devices.

We also point out that our use case is based on data from The Things Network (TTN), a global collaborative LoRaWAN network crowdsourced by enthusiasts and with more than 4000 gateways [22]. Because of the nature of this IoT network, many gateways are randomly deployed, particularly in urban areas. Furthermore, the community members are encouraged to build their own gateways and private deployments might use a variety of available options in the marketplace, from macro gateways to pico gateways, e.g., [23]. As a result, the coverage areas of gateways are heterogeneous and overlap each other with diverse signal strength values. For these reasons, LoRaWAN networks such as TTN may be deemed as HetNets.

## 4. Proposed Scheme

Since our proposed solution for the load balancing problem is an ML-aided scheme, our methodology is data-driven and divided into four main stages: data preprocessing, pattern analysis, classification method, and decision-making model. The following subsections provide the details about each phase.

### 4.1. Data Preprocessing

In this stage we gather the historical data from an actual operating network. As mentioned in Section 3, the use case for our method is an IoT LoRaWAN network and that is why we take advantage of the TTN initiative. Specifically, we use the data available at the TTN Mapper website [24]. The TTN Mapper is an application fed by users with mobile devices and its main objective is to map the TTN gateways coverage by sending UL packets. For this work, we use the data dumped into tab-delimited files.

**Figure 2:**
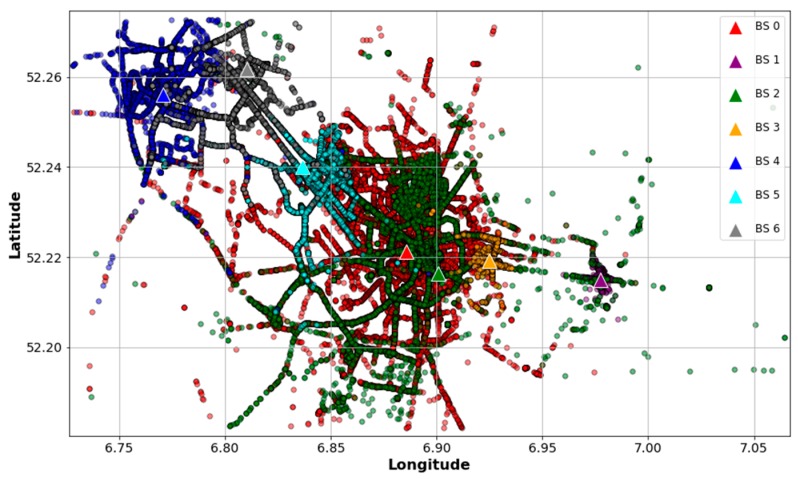
Locations of BS and their associated devices within the selected urban area.

**Figure 3:**
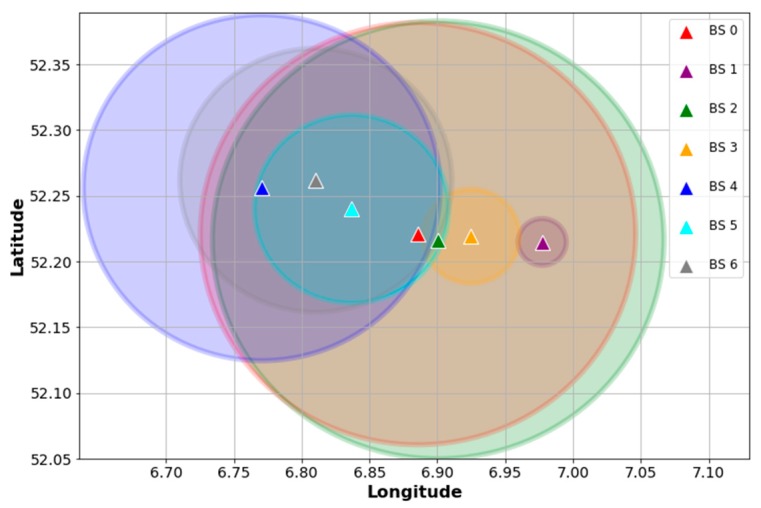
Coverage approximation of BS based on data points, assuming isotropic radiation, and ideal propagation.

Since the files contain raw data, the first step is to clean and select the entries that are useful for our problem. To this end, we searched for data corresponding to an urban area taking into account the following considerations: (1) the BS with the highest number of received packets is the reference BS; (2) other BS are selected within a 10 km radius of the reference BS; (3) as end devices are mobile, only entries with location information of devices are considered; and (4) every BS is associated with two or more devices, thereby avoiding “dedicated” BS in the analysis. The resulting data is a subset of 261,576 samples, corresponding to seven BS. Figure 2 depicts the locations of the found BS and their devices in order to visualize how they are distributed and associated. Similarly, Figure 3 shows an idealization of the BS coverage based on their associated devices’ locations. As can be seen, the data points show an urban scenario where some gateways behave like SBS and others like MBS. For example, BS 1 and BS 3 have shorter coverage ranges compared with the other gateways and their devices might be associated with BS 0 or BS 2, as well. Therefore, the selected data is suitable for our scheme and is consistent with our hypothesis of treating an urban dense IoT network as a HetNet.

Secondly, as our goal is to bias the device association to accomplish a load balancing, we extract several variables from data and waive the SNR and RSSI metrics. The main reason of doing so is to learn from the correlation among device’s variables that are not directly influenced by the signal strength values. Thus, the features to be analyzed are some already available in the dataset such as frequency, data rate, latitude, and longitude, and others extracted from the timestamp field like time of the day, and day of the week. The idea of using these variables is to learn from their values as they describe the particular situation of a device at the moment that is successfully transmitting a packet to the BS.

### 4.2. Pattern Analysis

The purpose of this phase is to find out whether the extracted features provide differentiated patterns for each BS. To this end, we analyze the samples of the seven BS by using the principal components analysis (PCA). PCA is an unsupervised ML technique widely used for data visualization and feature selection. PCA is a linear transform that maps the data into a lower dimensional space, known as the principal subspace, preserving as much data variance as possible, i.e., with minimum loss of information [25]. Since our features are frequency, data rate, latitude, longitude, time of the day, and day of the week, the original dimension of our data is a matrix N×D, where D=6 and N=261,576. In this way, our objective is to project the data of each BS into two dimensions, i.e., D=2, in order to visualize and verify that the extracted features do show a distinctive pattern. Therefore, for each BS there are $N_{k}$ samples and PCA will produce two vectors of $N_{k}$ elements, corresponding to the first two principal components. These vectors are computed as follows:

(1) $$p_{c}=w_{c}^{T}x_{k}$$

where c={1, 2}, $w_{c}$ are the projection vectors, and $x_{k}$ are the data subsets of each BS, i.e., k={0, 1, 2, 3, 4, 5, 6}. In this case, the learning task is to choose $w_{c}$ so that vectors $p_{c}$ have the maximum variance. Thus, PCA determines vectors $w_{c}$ by maximizing the variance in the projected space and by making them orthogonal, which means that $w_{1}^{T}w_{2}=0$. This maximization problem can be solved through the incorporation of Lagrangian terms [25], that yields:

(2) $$Sw_{c}=\lambda_{c}w_{c}$$

The pairs $\lambda_{c}$ and $w_{c}$ are the eigenvalues and the eigenvectors, respectively, of the covariance matrix S, which is defined by (3):

(3) $$S=\frac{1}{N_{k}}\overset{N_{k}}{\underset{n=1}{\sum }}\left(x_{k_{n}}-{\bar{x}}_{k}\right){\left(x_{k_{n}}-{\bar{x}}_{k}\right)}^{T}$$

where ${\bar{x}}_{k}$ is the mean of sample subset $x_{k}$.

Consequently, the variance will be maximum when $w_{1}$ is equal to the eigenvector with the highest eigenvalue $\lambda_{1}$, giving as result the first principal component. The second principal component is given by selecting a new direction, so that $w_{2}$ is orthogonal to $w_{1}$ and equal to the eigenvector with the second highest eigenvalue $\lambda_{2}$.

Finally, it is also important to highlight that before performing the PCA, each feature is normalized by using the min-max scaling method (4):

(4) $$z=\frac{x-x_{\min}}{x_{\max}-x_{\min}}$$

where *z* represents the normalized data points and *x* the original ones. The main objective of the scaling procedure is to have the values of all features within a range that is not too large, so that the variance maximization is not affected by their actual values [26]. Also, we have delimited $N_{k}=10,000$ in order to have an equal number of samples for each BS and make a fairer comparison among their patterns.

### 4.3. Classification Method for Association Biasing

In this stage, we use the data to train the system and determine a biased association between a device and a particular BS. In our use case, we denote the device-BS association as the selection of a BS to relay DL packets. We do this distinction as a LoRaWAN device may be connected to several gateways to send UL packets to the Network Server, so biasing in UL makes no sense and is not consistent with the standard. On the other hand, we assume that the default DL association in TTN is based on signal strength, as suggested in [27]. Then, our purpose is to bias that DL path configuration, recognizing that a bidirectional traffic in a LoRaWAN network represents a more realistic scenario [28].

To bias the device-BS association, we take advantage of the labeled data by applying a supervised learning technique. Specifically, this technique is intended to perform a multi-class classification, since our goal is to predict the BS that should forward DL messages to an end device by avoiding the SNR and RSSI metrics. Hence, in our use case scenario we have seven classes, one per BS. In addition, the inputs of the classifier are the features contemplated for PCA and the labels, which are categorical values corresponding to one of the seven classes.

ML classification algorithms can be categorized into two types: probabilistic and non-probabilistic classifiers. The main difference between them is that non-probabilistic classifiers define a decision boundary to determine whether a prediction belongs or not to a specific class [29]. It means that a non-probabilistic classifier performs a hard classification: given the inputs values, the model yields only one class. On the other hand, a probabilistic classifier provides the probabilities of belonging to each class, instead of giving only one class as a result. Then, a probabilistic classifier produces a soft classification and does not define decision boundaries. As we want to bias the default device-BS association, it is desirable to find the probabilities of receiving DL packets through other BS. For this reason, we choose to use a probabilistic classifier. Additionally, these kinds of classifiers allow us to find the classification posterior probabilities, which can be used for our decision-making problem of load balancing.

In general, probabilistic classifiers are based on the Bayes’ theorem to find the posterior class probabilities and determine the class membership for each new input x [25]. Thus, the posterior probabilities $p(C_{k}|x)$ are given by (5):

(5) $$p\left(C_{k}|x\right)=\frac{p\left(x|C_{k}\right)p\left(C_{k}\right)}{p\left(x\right)}$$

where $p\left(x|C_{k}\right)$ represents the class-conditional densities individually inferred for each class $C_{k}$, $p(C_{k})$ are the prior class probabilities, which can be estimated from portions of the training subset, and p(x) is found as follows (6):

(6) $$p\left(x\right)=\underset{k}{\sum }p\left(x|C_{k}\right)p\left(C_{k}\right)$$

To select a specific classification method, we compare the accuracy and the computational time of several algorithms, such as multiple logistic regression (MLR), Gaussian naive Bayes (GNB), Linear Discriminant Analysis (LDA), Quadratic Discriminant Analysis (QDA), and Decision Trees (DT). In addition, we include in our comparison some ensemble methods such as the Random Forests (RF), Extra Trees (ET) and a voting classifier. Details about the algorithms behind these classifiers can be found in [26,29,30].

Similar to the pattern analysis, an equal number of samples $N_{k}=10,000$ are extracted for each class in order to have a balanced dataset and prevent the classifiers from being biased during the training process. To train and test the classifiers, the dataset is divided into two subsets: 80% and 20%, respectively. Based on these subsets, we also calculate the average accuracy of each classifier. In this way, we determine the true positive (TP), true negative (TN), false positive (FP), and false negative (FN) classification outcomes per class by comparing the predicted labels to the actual labels from the test subset samples. Note that a hard classification is needed for this comparison, therefore, we consider the class with the highest probability as the predicted label. Accordingly, the terms TP, TN, FP, and FN are derived from the confusion matrix, which summarizes the comparison: columns describe the outputs of predicted labels and rows, the actual labels. Thus, the value of TP for class 1, for example, is the number of predictions with label 1 that match the actual label 1, and the number of predictions that do not match is the value of FP. Similarly, TN is the number of predictions with other label different from label 1 that match any other actual label, and FN represents the otherwise case. Subsequently, the overall classifier accuracy with K classes can be calculated by macro-averaging the accuracy of the classes [31], i.e., all classes equally treated, as follows (7):

(7) $$\mathrm{Accuracy}=\frac{1}{K}\overset{K}{\underset{k=1}{\sum }}\frac{{\mathrm{TP}}_{k}+{\mathrm{TN}}_{k}}{{\mathrm{TP}}_{k}+{\mathrm{TN}}_{k}+{\mathrm{FP}}_{k}+{\mathrm{FN}}_{k}}$$

Additionally, we point out that before training the classifiers, the features are standardized by using the z-score Formula (8):

(8) $$z=\frac{x-\mu }{\sigma }$$

where z is the standardized data point value, x is the original value, μ and σ are the mean and the standard deviation of each variable, respectively. In this way, the classifiers perform better with standard normally distributed data, i.e., with zero mean and unit variance [26].

Finally, we define $r_{k}$ as the vector with the found probabilities after making a prediction for the biased association. Therefore, the values of $r_{k}$ correspond to a device’s probabilities of being associated with specific BS by waiving the signal-based features, and then $\underset{k}{\sum }r_{k}=1$.

### 4.4. Decision-Making Model for Load Balancing

**Figure 4:**
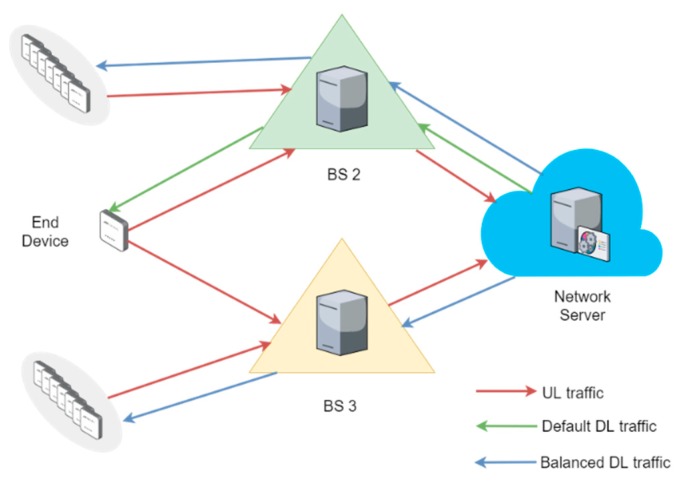
Example of a load-balancing decision to be made.

Our goal with the decision-making model is to achieve a load balance and, consequently, improve the network capabilities in terms of PDR and energy cost of data delivery. Without loss of generality, we delimit our analysis to those cases when an end device transmits UL packets to two BS at the same time. In this fashion, we filter the original dataset, obtaining a new subset with 17,146 samples. For example, a pair of samples from that subset represent an end device that concurrently sends UL traffic to BS 2 and BS 3, as shown in Figure 4. The default path for DL traffic depends on the BS with the highest RSSI, as we explained in Section 4.3. In our example, that default DL association is done via BS 2. Therefore, the decision to be made is whether DL packets are forwarded through the BS corresponding to the default path or not. In the latter case, the DL traffic would be transferred to BS 3. As mentioned in Section 2, our decision-making model is based on an MDP, so that the Network Server can make decisions on DL load balancing at each BS. We also model our MDP with some calculations based on data of the new subset.

Generally speaking, an MDP is a sequential decision problem for an observable and stochastic environment with the Markovian property. In other words, MDPs are a fundamental formalism for sequential learning problems in stochastic domains, such as decision-theoretic planning and reinforcement learning [32]. A set of states s, a set of actions in each state a(s), the transition probabilities among states $P(s^{\prime }|s,a)$, and a reward function R(s) comprise an MDP. Thus, a decision maker (also known as agent) must choose to perform an action when the process is in a singular state, based on a policy π, which is the decision solution given P and R [33]. For our scheme, we model an MDP with states corresponding to the number of BS. There are two actions to complete in each BS: to offload or not to offload its DL traffic, i.e., a(s)={0, 1}. Two matrices describe the values of P for each action, defined as $P_{0}$ when the decision is to not offload, and $P_{1}$ to offload the BS.

We assume that any device is concurrently transmitting confirmed packets to two BS, which means that one of those BS must respond an ACK, i.e., a DL message. As we explained in Section 4.3, the default DL association for transmitting an ACK is between the BS with highest RSSI and the end device. Therefore, the decision that the Network Server has to make is whether the DL association remains with the default BS, a(s)=0, or switches to the other one, a(s)=1. Subsequently, the probability of being in state s and staying in that state if the decision is to not offload is $P\left(s^{\prime }=s|s,a=0\right)=1$, and then $P_{0}$ is defined as follows (9):

(9) $$P_{0}=\left[\begin{array}{ccccccc} 1 & 0 & 0 & 0 & 0 & 0 & 0\\ 0 & 1 & 0 & 0 & 0 & 0 & 0\\ 0 & 0 & 1 & 0 & 0 & 0 & 0\\ 0 & 0 & 0 & 1 & 0 & 0 & 0\\ 0 & 0 & 0 & 0 & 1 & 0 & 0\\ 0 & 0 & 0 & 0 & 0 & 1 & 0\\ 0 & 0 & 0 & 0 & 0 & 0 & 1 \end{array}\right]$$

To calculate $P_{1}$, the transition probabilities can be estimated from historical records [34]. Thus, we use the data samples to count the total number of device associations that each BS had and the shared associations between each pair of BS. Hence, the probabilities that the DL traffic is offloaded from a BS to another BS, $P\left(s^{\prime }|s,a=1\right)$, are given by (10):

(10) $$P_{1}=\left[\begin{array}{cccc} 0 & \frac{\sum \left(x_{0}\cap x_{1}\right)}{\sum x_{0}} & \ldots & \frac{\sum (x_{0}\cap x_{6})}{\sum x_{0}}\\ \frac{\sum \left(x_{1}\cap x_{0}\right)}{\sum x_{1}} & 0 & \ldots & \frac{\sum (x_{1}\cap x_{6})}{\sum x_{1}}\\ \vdots & \vdots & \ddots & \vdots \\ \frac{\sum \left(x_{6}\cap x_{0}\right)}{\sum x_{6}} & \frac{\sum \left(x_{6}\cap x_{1}\right)}{\sum x_{6}} & \ldots & 0 \end{array}\right]$$

With respect to R(s), we also estimate its values based on the historical data and the classifier results. Basically, we compute how busy a BS might be transmitting DL packets to define how “rewarding” that BS is. In this way, the more occupied a BS is, the higher its reward is for the offloading decision. This consideration is consistent with the fact that gateways utilization is taken into account to schedule DL traffic in TTN [27]. Then, the rewards vector for the MDP is calculated as follows (11):

(11) $$R=\overset{N_{A}}{\underset{n=1}{\sum }}r_{k_{n}}$$

where $N_{A}$ is the total number of end devices requesting ACKs and $r_{k}$ is the vector containing the obtained probabilities from the classifier.

It is also important to point out that we model our MDP with an indefinite horizon for the decision making, which means that there is no fixed time limit and that the optimal policy $\pi^{*}$ is stationary [33]. Also, we consider a discount factor γ that describes the preference of the decision maker (in our case, the Network Server) for current rewards over future rewards. Accordingly, the utility of a state sequence is defined as (12):

(12) $$U=\underset{k}{\sum }\gamma^{k}R\left(s_{k}\right)$$

More importantly, we must find $\pi^{*}$ for our MDP, which is an optimization problem to choose the action that maximizes the expected utility of the subsequent state (13):

(13) $$\pi^{*}\left(s\right)={\mathrm{argmax}}_{a}\underset{s^{\prime }}{\sum }P\left(s^{\prime }|s,a\right)U\left(s^{\prime }\right)$$

There are several methods to solve this optimization problem. In this work, we assess the performance of two well-known algorithms: value iteration and policy iteration. On the one hand, the value iteration algorithm calculates the utility of each state and then iteratively uses the state utilities to select an optimal action in each state. On the other hand, the policy iteration algorithm alternates between the evaluation of the states utilities under the current policy (starting from some initial policy) and the improvement of the current policy with respect to the current utilities. Details about these and other algorithms can be found in [33,34].

**Figure 5:**
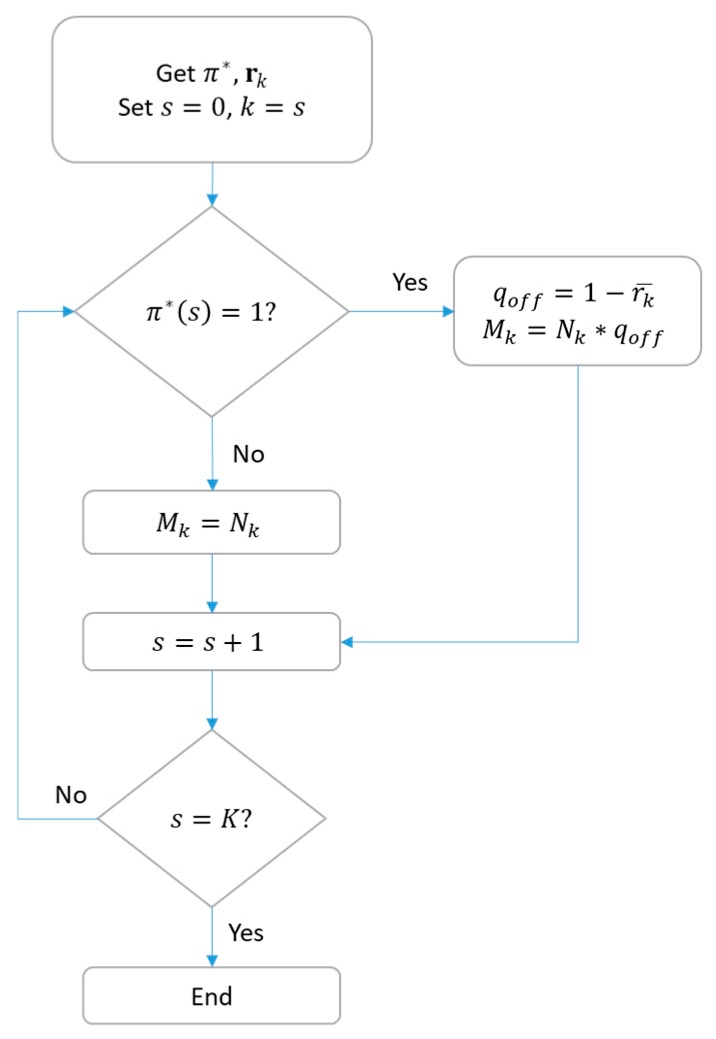
Algorithm for the traffic offloading decision.

Another point to consider is that we define the quantity of DL traffic offloading based on the classifier outputs to avoid that any BS ends up with no packets to transmit. As a result, the quantity of end devices to be offloaded from a BS, that is $M_{k}$, depends not only on $\pi^{*}$ but also on $r_{k}$, as shown in Figure 5. In this flowchart, ${\bar{r}}_{k}$ is the mean value of vector $r_{k}$, $N_{k}$ is the number of devices associated with a specific BS, and K is total number of BS in the network.

## 5. Network Simulation Design

To simulate a system using our proposed scheme, we adapt some analytical models found in the literature for the simulation of LoRaWAN networks. As we assume that in the network all the devices are Class A, they use the uncoordinated transmission scheme ALOHA. The PDR in a network that employs pure ALOHA can be modeled based on a Poisson distribution [35], as follows (14):

(14) $$\mathrm{PDR}=e^{-2N*T_{Packet}*\lambda }$$

where N is the number of devices in the network, $T_{Packet}$ is the average airtime that takes transmitting a packet, and λ is the average packet arrival rate. However, this model does not take into account the retransmissions when devices request ACKs from the network. Therefore, the model is adapted to consider the retransmissions (15):

(15) $${\mathrm{PDR}}_{A}=e^{-2N_{A}*T_{Packet}*\lambda *p_{BS}}$$

$N_{A}$ is the total number of end devices requesting ACKs, as described in Section 4.4, and the new term $p_{BS}$ is the blocking probability of a BS due to the ACKs (DL traffic), given by (16):

(16) $$p_{BS}=1-{\left(1-q_{BS}\right)}^{A_{Tx}}$$

where $q_{BS}$ is the ratio between DL traffic and UL traffic in a BS and $A_{Tx}$ is the number of retransmissions of a device before receiving an ACK. As the LoRaWAN standard specifies a maximum number of seven retransmissions and considering the original transmission as a retransmission, according to [36], we run our simulations with $A_{Tx}=8$, which corresponds to the worst case. It is also important to highlight that, for the $q_{BS}$ calculation, the DL traffic is either the default load or the balanced load at the BS, depending on the offloading decision.

Next, to simulate the PDR over all the BS in the network, we calculate the total PDR following the product form (17):

(17) $${\mathrm{PDR}}_{\mathrm{Total}}=\overset{K}{\underset{k=1}{\prod }}{\mathrm{PDR}}_{A_{k}}$$

where K is the total number of BS, that is K=7 for our use case scenario.

In our simulations, each experiment represents an MDP. For each experiment, we take samples from the subset described in Section 4.4. In this manner, we conduct more realistic experiments by using actual data instead of synthetic data. An experiment consists of randomly selecting a pair of samples corresponding to an end device associated with two BS. Without loss of generality, we delimit our analysis to $N_{A_{\max}}=5000$, starting with an experiment of 5 devices and increasing the number by 5 in each experiment. In relation to $T_{Packet}$, we choose an airtime that is consistent with common LoRaWAN deployments like TTN. Consequently, we set $T_{Packet}=1712.13\;\mathrm{ms}$, which is a robust packet airtime for those kinds of deployments, according to [37].

With respect to the energy cost of data delivery (ECD), we adapt the model for a dense LoRaWAN network presented in [38]. Thus, the ECD is given by (18):

(18) $$\mathrm{ECD}=\alpha \frac{e^{N_{A}*\lambda *p_{BS}*L_{Pl}}}{L_{Pl}}$$

where α is a constant expressed in Joules and $L_{Pl}$ is the size of messages payload. We assume that a typical Smart City IoT application transmits messages with a payload size of 20 bytes, on average. For both PDR and ECD models, Table 1 summarizes the parameters used in our simulations.

## 6. Evaluation Results

We evaluate our method through computer simulations and, specifically, by running code written in Python 3. Some packages for data analysis and ML are used, such as pandas [39], scikit-learn [40], and MDPToolbox [41]. The simulations are run on a PC with Ubuntu 16.04 64 bits, processor Intel^®^ Core™ i3 CPU M 350 @ 2.27 GHz × 4, and RAM of 4 GB. Note that we decided not to use High Performance Computing systems, as we are aware that many private LoRaWAN deployments do not count on these sorts of resources. In the following subsections we present the numerical results of our simulations and discuss their implications.

### 6.1. PCA Patterns

**Figure 6:**
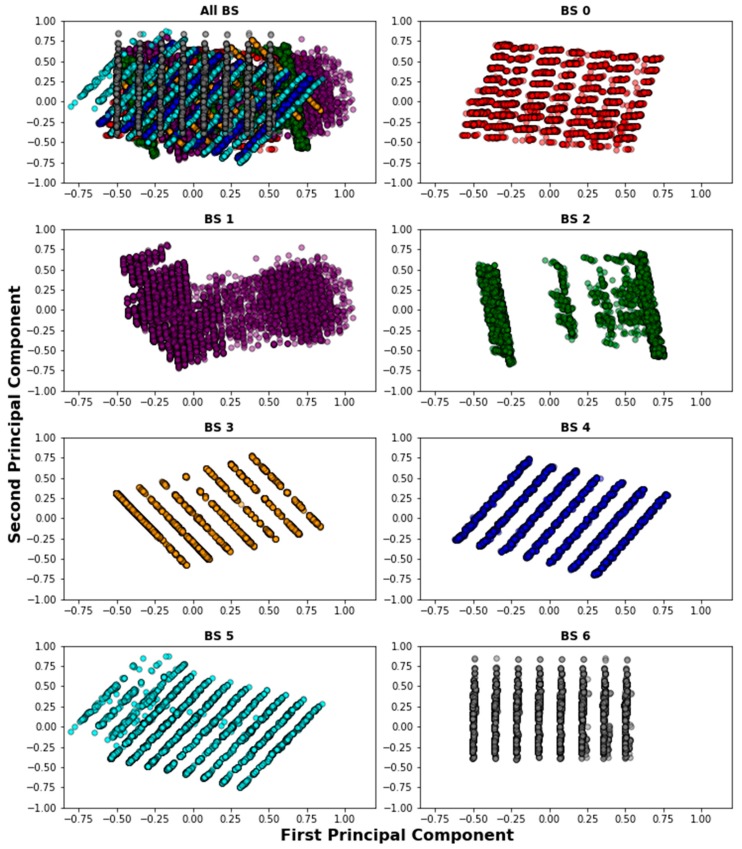
Discovered patterns for BS after projecting the first two principal components.

As explained in Section 4.2, we want to discover whether there is a characteristic pattern for each BS when the device association is biased by obviating the RSSI and SNR metrics. Therefore, the PCA analysis is performed taking into account the normalized values of the features: frequency, data rate, latitude, longitude, time of the day, and day of the week. To visualize the analysis, Figure 6 shows the pattern projected by the first two principal components for each BS. It is noticeable that each BS depicts a different pattern, which means that a device with particular values of the extracted features might be associated with specific BS through classification. In other words, we can predict the device’s probability of having a specific DL path by biasing the signal-based variables.

### 6.2. Classifiers Outcomes

**Figure 7:**
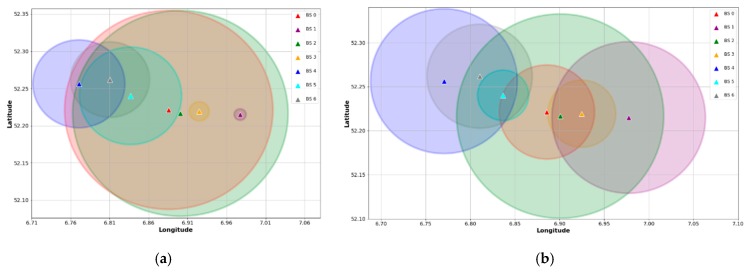
Device-BS association comparison: (**a**) estimated coverage based on RSSI; (**b**) CRE based on biased association.

As we explained in Section 4.3, our goal with the classifier is to bias the default device-BS association based on signal strength measurements like RSSI. Then, the classifier is trained with features which represent the particular condition of the devices, excluding the signal-based variables. To evaluate the association biasing, we use data samples from the subset described in Section 4.4. In this manner, we select 8500 devices that simultaneously transmit UL packets to two BS. Assuming that the default association is given by the BS with the highest RSSI, an approximation of all BS coverage is shown in Figure 7a. As can be seen, this coverage mapping is comparable to that depicted in Figure 3. On the contrary, Figure 7b illustrates a coverage map estimate based on the classification results. In this case, the association of a device with the default BS is changed to the BS with the highest probability given by the classifier. It is noticeable that the proposed biasing method yields a CRE, as described in Section 2. For instance, the range of BS 1 and BS 3, which act as SBS, are virtually expanded after performing the association biasing.

**Figure 8:**
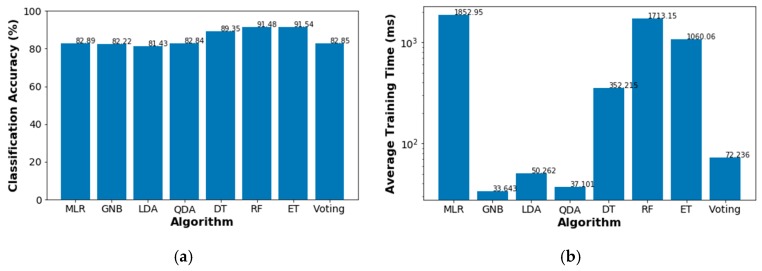
Classifiers performance comparison: (**a**) average classification accuracy; (**b**) average training time.

To compare the performance of the probabilistic classifiers, we ran the training code 5000 times. Figure 8 shows the average classification accuracy and the average training times for each considered algorithm. The voting method is an ensemble classifier that combines the classification results from GNB and QDA. As can be seen, the most accurate classifier is ET, however, this algorithm also employs the third longest training time. In contrast, our intention with the voting classifier is to evaluate any accuracy enhancement given by the combination of the two fastest algorithms, i.e., GNB and QDA. Although the accuracy of the voting algorithm is slightly above the QDA’s score, the total training time is approximately the summation of their individual training times. For these reasons, we finally use the ET algorithm outcomes as inputs for the decision-making model.

### 6.3. Network PDR Improvement

In this subsection we present the obtained results when the network is simulated with the parameters specified in Section 5. We first compare the PDR improvement achieved with our proposed scheme through two simulation setups: MDP with and without the classifier. The objective is to determine if the combination of the classification method and the modeled MDP really makes a difference compared to the MDP working alone. Note that, to compare the MDP results without the classifier, the reward vector R is found by counting the number of default DL associations. In this way, one simulation setup is based on the association biasing given by the classifier’s predictions (as described in Section 4.4) and the other setup relies on the RSSI-based association.

**Figure 9:**
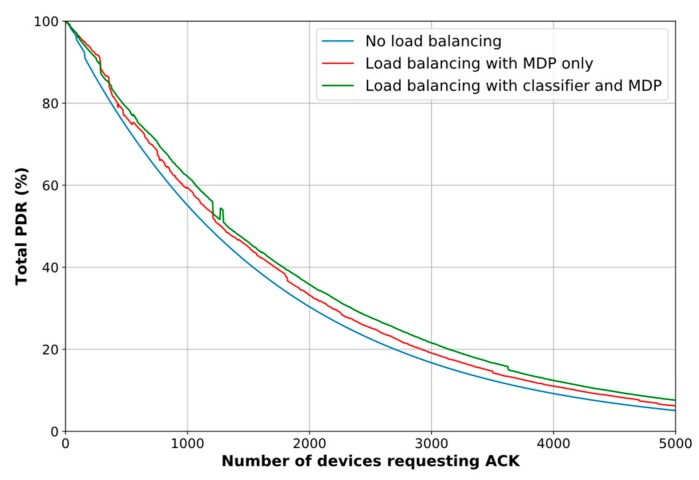
Network PDR improvement based on proposed scheme.

**Figure 10:**
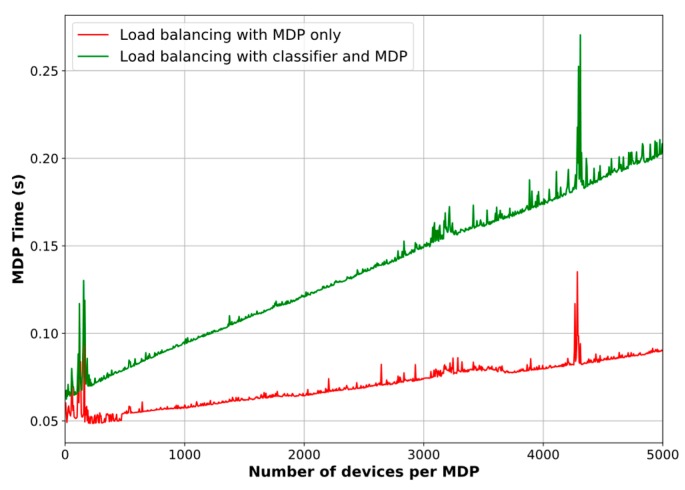
Computational time comparison for the MDPs.

Figure 9 depicts the resulting graphs of the system simulation in terms of PDR. As can be seen, the proposed scheme performs better when the outcomes from the classifier are taken into account, particularly in the circumstances when many devices are requesting DL traffic (note that the MDP-only load balancing method outperforms the combined method just when the number of devices is small, i.e., between 0 and 300 devices, roughly). However, there is a trade-off between the PDR improvement and the computational time, Figure 10. We point out that in this comparison we only consider the classifier’s prediction time, in other words, we do not include its training time, as we assume that the Network Server has previously trained the model. It is noticeable that when the proposed scheme uses the association biasing based on the predicted classes, the MDP needs more time to make a decision on load balancing. It is also important to highlight that the graph show some peaks, which means that the iteration algorithm employed more iterations to find $\pi^{*}$. Because of the stochastic nature of the samples, the algorithm might have dealt with tough values to determine $\pi^{*}$. However, we can see that in those cases, although more time was needed, the goal of improving the PDR was achieved.

In terms of improvement percentages, we find that the PDR increases by 13.11%, on average, and up to 26.8% without the classifier. Similarly, the PDR rises by 23.74%, on average, and up to 49.98% when the classifier results are incorporated in the decision-making model. In contrast, the average decision time is 89.33% higher for the latter case, reaching a maximum of 0.27 s. However, we highlight that the decision process is run on the Network Server which is supposed to have enough resources to deal with this trade-off and take advantage of a better PDR for the whole network.

Additionally, as mentioned in Section 4.4, we compare the computational time of both value iteration and policy iteration algorithms to solve the MDPs. The measured average decision times are 70 ms and 95 ms for the policy iteration and the value iteration methods, respectively, after running the experiments with the MDP-only simulation setup. This fact reveals that the policy iteration method is about 26% faster than the value iteration method to find the optimal policy of our load balancing decision model. That is why we used the policy iteration algorithm for the comparison described in Figure 10.

### 6.4. Network ECD Reduction

**Figure 11:**
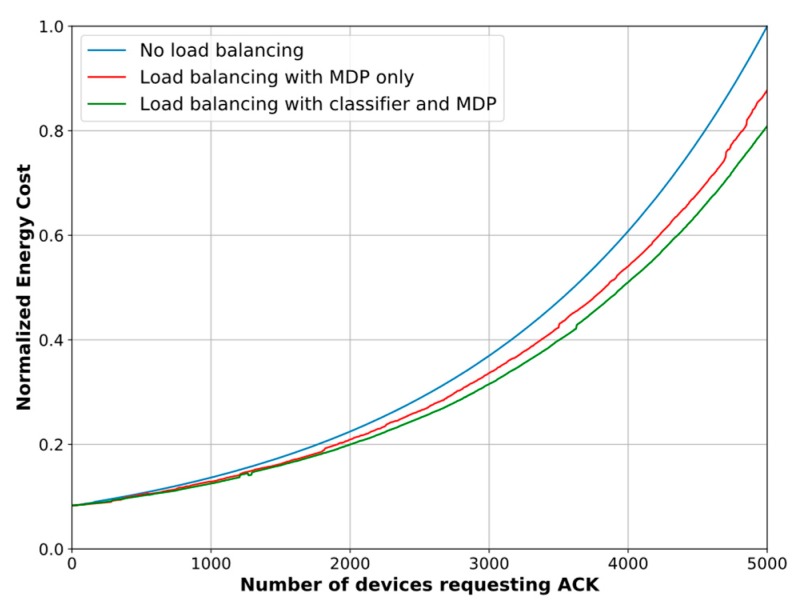
Network EDC reduction based on proposed scheme.

In relation to the ECD, we also compare the results of the MDPs with and without the association biasing. Figure 11 depicts the normalized ECD. Similar to the PDR evaluation results, our proposed scheme yields an ECD reduction of 8.1%, on average, and up to 13.36% when the classification method is ignored. Conversely, a maximum reduction of 19.1% and an average ECD reduction of 12.04% are achieved when the biasing method, based on the classifier, is included in the load balancing model.

## 7. Conclusions

In this work, we proposed a scheme for load balancing in HetNets that can be applied to dense IoT networks such as Smart Cities scenarios. Our approach is based on several ML techniques to discover hidden patterns (PCA), learn from the labeled data (supervised probabilistic classifiers) and make decisions (MDP). As a use case, we validated our method with data from an actual LoRaWAN IoT network. Once we preprocessed the data, we confirmed that such a network deployed in urban areas can be deemed as a HetNet.

We demonstrated that with our scheme the goal of device-BS association biasing can be achieved based on predictions that are made by obviating signal-based features. Unlike other related works, we used labeled data for biasing the device-BS association through a supervised classifier. This approach solves the CRE problem in such a manner that is less complex to implement than other solutions based on reinforcement learning. Therefore, the proposed association biasing method might be more suitable in scenarios where the computational resources of core network elements, such as the Network Server, are more constrained.

We also confirmed that our MDP-based decision-making model for the traffic offloading has better results when the classifier’s predictions are considered. The evaluation results describe the improvement of network capabilities in terms of PDR (an increase of 50%) and reduction of ECD (nearly a decrease of 20%). On the other hand, although MDPs are the basis for reinforcement learning algorithms such as Q-learning, our method does not consider the action-value function Q(s,a), i.e., the current state and each possible action that can be taken individually. In other words, in our method the policy and expected reward are based on the current state and the average across all of the actions that can be taken. Therefore, our method needs less data as the function Q is not considered. However, in the future we will study the trade-off of getting better results by including Q and the likely longer time to learn and make decisions. This is also a relevant consideration for wireless networks with very restricted resources.

In this paper, we validated our methodology through a specific standard, but the method may be implemented in IoT networks operating under other standards, particularly in dense environments. It is also important to highlight that the only process of our scheme that runs in real time is the MDP and, consequently, the data preprocessing and the classification training can be carried out offline. However, in future implementations we recommend performing these tasks periodically (not necessarily in real time) in order to obtain more accurate results thanks to the updated data.

Although the time delay caused by the decision process of our model may be unacceptable for several WAN RAT applications, we point out that a particular characteristic of technologies like LoRaWAN is that they are focused on the connectivity of devices that transmit messages in relatively long periods at low data rates. Nevertheless, more research is needed about the optimization of methods like the one we have proposed, as well as the time complexity analysis for their implementation in specific solutions, especially where there is a large number of end devices.

As a future work, the simulations could be run on a discrete-event simulator to compare the results to those presented in this paper. Furthermore, an even more realistic scenario may be set such as a prototype network with a server running our scheme and a significantly large number of tiny devices using its services. Additionally, the possibility of combining some of other techniques described in the literature with ours might be explored, in order to obtain better results in terms of energy efficiency.

Finally, thinking of a LoRaWAN network specifically, we point out the importance of considering more adjusted parameters such as data rate, number of retransmissions, and packet arrival rate. Since we used values corresponding to worst-case scenarios in our analytical models, better results can be achieved with our scheme by adjusting those variables to specific situations.

## Figures and Tables

**Table 1:** Simulation parameters for the evaluation of Packet Delivery Ratio and Energy Cost of Data Delivery.

Parameter	Description	Value
γ	Discount factor for MDP	0.9
$N_{A}$	Number of end devices requesting ACK	{5, 10, 15, ⋯ 5000}
λ	Average packet arrival rate	0.25 × 10^−4^ packets/ms
$T_{Packet}$	Packet airtime	1712.13 ms
$A_{Tx}$	Number of retransmissions	8
α	Energy constant	0.4 J
$L_{Pl}$	Size of messages payload	20 B
